# Timed and targeted transfusion in Rh isoimmunization: A case report on early detection and neonatal outcomes

**DOI:** 10.5339/qmj.2026.64

**Published:** 2026-09-22

**Authors:** Sameena Haroon, Muskan Aggarwal, Haroon Hussain, Adarsh Sugathan, Shamathmika Rao, Nayanatara Arun Kumar

**Affiliations:** 1 Department of Obstetrics and Gynaecology, Kasturba Medical College, Mangalore, Manipal Academy of Higher Education, Manipal, India; 2 Department of Medicine, Kasturba Medical College, Mangalore, Manipal Academy of Higher Education, Manipal, India; 3 Department of Internal Medicine, Manipal Hospitals, KMC Hospital, Jyothi Circle, Mangalore, Karnataka, India; 4 Department of Neuromicrobiology, National Institute of Mental Health and Neurosciences (NIMHANS), Bengaluru, India; 5 Department of Physiology, Kasturba Medical College, Mangalore, Manipal Academy of Higher Education, Manipal, India

**Keywords:** Rh-isoimmunization, fetal anaemia, intrauterine transfusion, exchange transfusion, high-risk pregnancy, India

## Abstract

**Background:**

Rhesus (Rh) alloimmunization remains an important cause of fetal anaemia and perinatal morbidity despite the widespread use of anti-D immunoglobulin, particularly in regions with inadequate antenatal surveillance. Early detection of fetal anaemia using middle cerebral artery (MCA) Doppler ultrasonography and timely intervention are essential for improving neonatal outcomes.

**Case presentation:**

We report the case of a 35-year-old multigravida (G3P2L1) with Rh-negative blood group, managed at a tertiary-care referral centre in India. She had a previous history of neonatal death attributed to complications of Rh-isoimmunisation. During her current pregnancy, high antibody titres were detected at 32 weeks of gestation. MCA Doppler revealed severe fetal anaemia, following which a single intrauterine transfusion was performed at 33 weeks. Delivery was performed by caesarean section at 35 weeks and 4 days of gestation. The neonate required two double-volume exchange transfusions and intensive phototherapy in the immediate postnatal period. At 45 days of life, the infant developed late-onset normocytic normochromic anaemia, which was successfully managed with packed red blood cell transfusion.

**Discussion:**

This case highlights the importance of late third-trimester detection of severe fetal anaemia using MCA-peak systolic velocity (PSV) Doppler assessment, enabling timely intrauterine correction with a single transfusion and reducing the need for repeated invasive procedures. Despite antenatal optimization, the neonate required intensive postnatal management, including double-volume exchange transfusions, intravenous immunoglobulin (IVIG), and phototherapy, highlighting the continued impact of circulating maternal antibodies. Long-term haematological follow-up remained necessary.

**Conclusion:**

Early Doppler-based diagnosis, timely single intrauterine transfusion, structured neonatal intensive care, and long-term hematologic follow-up resulted in favourable survival with normal developmental progression at 15 months of age.

## 1. INTRODUCTION

Rh alloimmunization is an immune-mediated condition that occurs when an Rh-negative mother is exposed to positive fetal red blood cells, resulting in the formation of maternal anti-D antibodies.^1^ These antibodies can cross the placenta in subsequent pregnancies and cause haemolysis of fetal erythrocytes, leading to fetal anaemia, hydrops fetalis, or intrauterine fetal demise.^2^

Although the routine administration of anti-D immunoglobulin has significantly reduced the incidence of Rh-isoimmunization^3^ in developed healthcare systems, isolated cases continue to be encountered, particularly in low- and middle-income countries such as India, where access to standardized antenatal care may vary. Management of these pregnancies requires specialized fetal surveillance and availability of advanced obstetrics and neonatal care.

Fetal anaemia is currently assessed non-invasively using middle cerebral artery (MCA) peak systolic velocity (PSV), expressed as multiples of the median (MoM) for gestational age. In fetal anaemia, the peak velocity increases as a result of reduced viscosity and an elevated cardiac output. Importantly, there is no significant correlation between fetal haemoglobin concentration and MCA-PSV in cases of mild or no anaemia. However, as the haemoglobin level decreases, the MCA-PSV shows a corresponding rise, providing a reliable estimation method for assessing haemoglobin levels in the fetus. An MCA-PSV value exceeding 1.5 MoM^2,4^ reliably predicts moderate to severe fetal anaemia. The criteria also include a haematocrit of less than 30%.^1,5^ Intrauterine transfusion has become the cornerstone of management of severe fetal anaemia, significantly improving survival rates [6] when performed in specialized centres. A final transfusion at not more than 34 to 35 weeks is preferred by most clinicians, with the delivery targeted at 37 to 38 weeks.^1,4^ This case report describes the management of high-risk, Rh alloimmunized pregnancy at a tertiary care centre in India, emphasizing the importance of timely detection, judicious use of intrauterine transfusion, and long-term neonatal follow-up.

## 2. CASE PRESENTATION

A 35-year-old female from Karnataka with AB negative blood group presented to the Department of Obstetrics and Gynaecology, Manipal Hospitals, Mangalore, India. at 32 weeks of gestation with a significant obstetric history: gravida 3, parity 2, with 1 living child and 1 reported death. The corrected gestational age (GA) of the fetus was 32 weeks 3 days, conceived spontaneously, and the patient reported adequate fetal movements. Her menstrual history indicates that her last menstrual period was on 6/7/23, allowing us to accurately calculate an estimated due date of 11/4/24 (according to menstrual dates). Her past medical and surgical history was otherwise unremarkable, with no history of diabetes mellitus, hypertension, autoimmune disorders, or previous blood transfusions. Her husband is Rh positive (B-positive).

Upon examination, the patient’s vital signs were stable at a blood pressure of 110/70mm Hg, heart rate of 70 bpm, and SpO2 98%. A thorough abdominal examination revealed a uterus (UT) consistent with 32 weeks of gestation, positive external ballotment (EB), and fetal heart sounds (FHS) indicating the fetal condition is reassuring. Laboratory investigations confirmed an Rh-negative blood group (AB negative) with a positive indirect Coombs test, a haemoglobin level of 11.5g/dL, a total leukocyte count (TLC) of 6700, and a platelet count (PTL) of 179000/cc. Maternal antibody screening demonstrated isolated anti-D alloimmunization with no clinically significant additional red-cell antibodies detected. Diagnostic assessments through ultrasonography (USG) indicated a gestational age of 32 weeks 3 days, with an amniotic fluid index (AFI) measuring 13.8 and an estimated fetal weight (EFW) of 1955 grams.

### 2.1. Past obstetric history

Her first pregnancy, approximately eight years earlier, was uneventful and resulted in a healthy female neonate weighing 2.7 kilograms delivered vaginally. She received 300 micrograms of anti-D immunoglobulin postpartum.

Her second pregnancy, approximately three years earlier, was complicated by fetal intraventricular haemorrhage. She presented at 28 weeks of gestation with a positive indirect Coombs test. She was monitored closely, and fetal surveillance was done. A male baby weighing 3.03 kilograms with a good Apgar score was delivered by a lower segment caesarean section. Magnetic resonance imaging (MRI) revealed grade IV germinal matrix haemorrhage. Despite intensive phototherapy and close follow-up, the child died at 18 months of age due to complications related to severe neurological injury.

### 2.2. Present pregnancy

Given the previous adverse outcome three years earlier, the present pregnancy was managed as a precious pregnancy. She was booked for follow-up at 13 weeks of gestation, and serial indirect Coombs tests were performed every four weeks. Initial antibody screens were negative.

**Figure 1.**
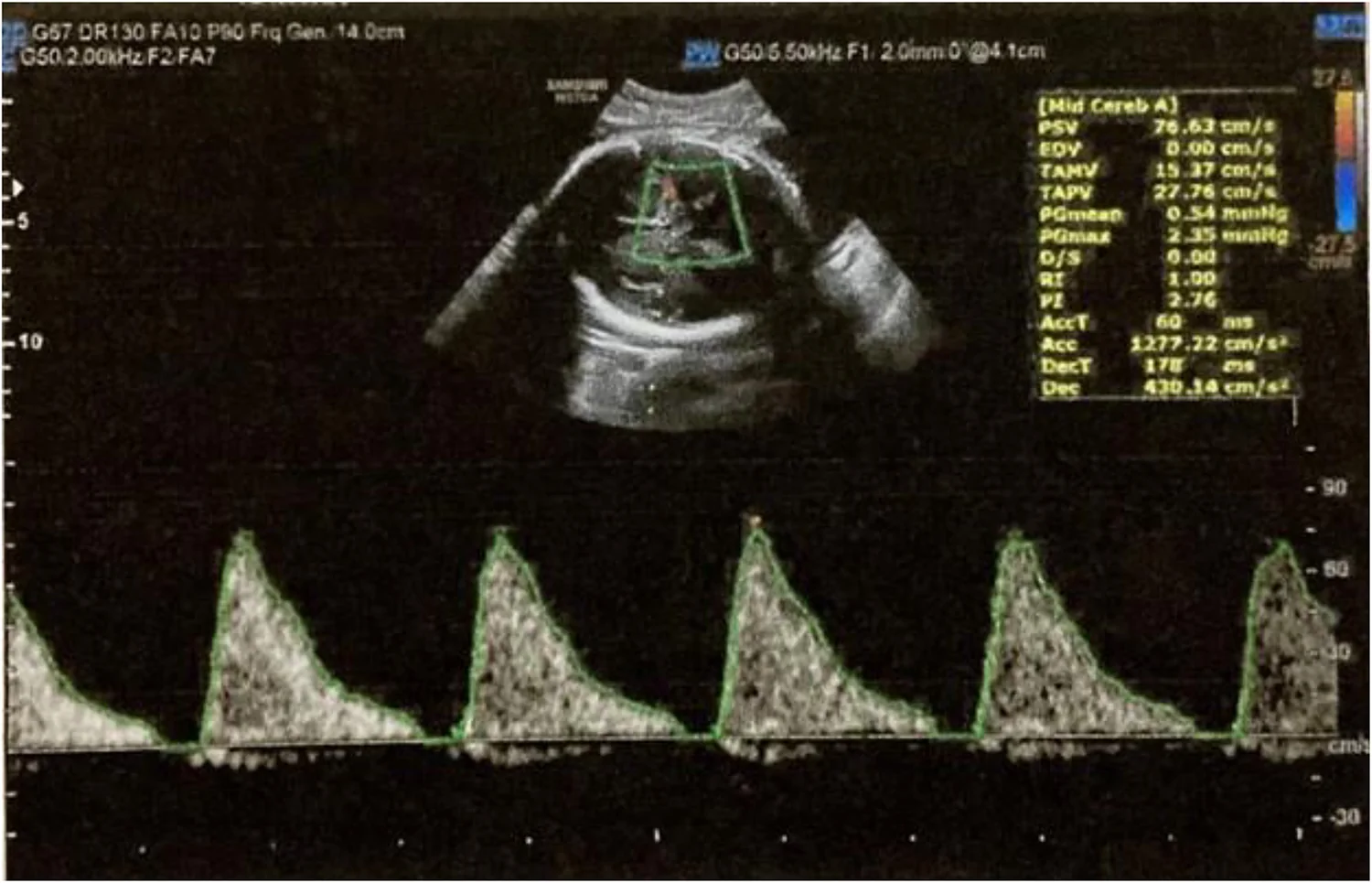
Middle cerebral artery (MCA) Doppler ultrasound demonstrating elevated peak systolic velocity of 76.6 cm/s (1.69 multiples of median), consistent with severe fetal anaemia.

Ultrasonography with Doppler at 19 and 28 weeks showed no evidence of fetal anaemia. However, at 32 weeks of gestation, the indirect Coombs test became positive with a high antibody titre of 1:256. MCA Doppler revealed a peak systolic velocity of 76.6 cm/s, corresponding to 1.69 MoM, indicating severe fetal anaemia (Figure 1).

### 2.3. Intrauterine transfusion (IUT)

A single intrauterine transfusion was planned at 33 weeks of gestation. The patient received thorough counselling regarding the intrauterine transfusion (IUT), emphasizing its importance for fetal well-being. An estimated fetal weight (EFW) of 1955 grams was noted prior to transfusion.

All aseptic precautions were taken. Guided by ultrasound, a 20-gauge needle was expertly inserted into the umbilical vein at the placental insertion site, from which a 0.5 mL fetal blood sample was obtained for analysis. The pre-procedure haematocrit was noted to be 16.5%, while the target haematocrit was calculated at 30%. Donor haemoglobin and haematocrit were 24.6 and 74%, respectively. A total of 80 ml of irradiated, Cytomegalovirus (CMV)-negative, leucodepleted, HbS-negative O-negative packed red blood cells were transfused into the umbilical vein, all under continuous ultrasound supervision. Injection Vecuronium Bromide (0.1 ml/kg body weight) was used for fetal paralysis and sedation. Maternal medications were administered according to institutional protocol.

Following the procedure, audible fetal movements and normal cardiac activity were reassuring indicators of fetal health. Subsequent ultrasound revealed an MCA-PSV of 59.91 cm/sec, with a multiple of the median (MoM) of 1.22 and a fetal heart rate (FHR) of 129 beats per minute. Post-transfusion fetal haemoglobin increased to 10.1 g/dL, and the haematocrit improved to 33.3%.

### 2.4. Delivery and neonatal course

Elective caesarean section was performed at 35 weeks and 4 days after multidisciplinary discussion because of the increasing risk of recurrent fetal anaemia following the final intrauterine transfusion, previous caesarean delivery, and institutional protocol favouring delivery after fetal lung maturity rather than repeat late-gestation transfusion. The male neonate weighed 2.06 kg and cried immediately after birth, with an Apgar score of 8 at 1 and 5 minutes. Intraoperatively, the baby had meconium-stained liquor, with umbilical cord wrapped around the neck. The neonate developed respiratory distress and was admitted to the Neonatal Intensive Care Unit (NICU), where continuous positive airway pressure (CPAP) was provided by nasal bubble for 47 hours.

Cord blood analysis revealed severe anaemia (haemoglobin 5.1 g/dL, haematocrit 16.4%) and hyperbilirubinemia (with peak bilirubin of 15mg/dl) in the exchange transfusion range. Blood group of the baby was B-positive, and the direct Coombs test was negative. An intravenous (IV) peripheral line was inserted under aseptic technique. IV fluids and antibiotics were initiated. An umbilical venous catheter was inserted, and a double volume exchange transfusion was performed using leucodepleted O-negative whole blood. Persistent hyperbilirubinemia necessitated a second exchange transfusion within 24 hours. A total of 420 mL of blood was exchanged. IVIG and intensive phototherapy were administered.

Feeding was gradually established, and the neonate showed steady clinical improvement. On day four of life, injection 10% calcium gluconate IV was given for 72 hours, in view of hypocalcaemia. The neurosonogram was within normal limits.

Repeat blood tests were normal. The baby was reunited with the mother on day five of birth after stabilization. He was discharged after fourteen days of birth with a haemoglobin concentration of 15.7g/dL.

### 2.5. Follow-up

At 45 days of life, the infant was brought back to the hospital with marked pallor. Investigation revealed normocytic normochromic anaemia (haemoglobin 6.4 g/dL and haematocrit 19.8%). 50 millilitres of O-negative packed red blood cells were transfused intravenously at a controlled rate of 10 mL per hour over five hours under continuous monitoring. A good haematological response was achieved. The infant has since been followed monthly for six months and every three months thereafter. He remains clinically stable and developmentally appropriate at 15 months of age.

## 3. DISCUSSION

Severe fetal anaemia in Rh immunised pregnancies often requires serial IUT at 2-to-3-week intervals depending on disease severity.^1^ However, in the present case, anaemia detected at 32 weeks was successfully managed with a single well-timed IUT at 33 weeks, suggesting that late gestation presentation with close surveillance may allow a less intensive transfusion strategy in selected patients. Pasman et al.^2^ reported MCA Doppler to be the gold standard for detecting fetal anaemia in alloimmunized pregnancies. This technique allows for non-invasive assessment, eliminating the use of amniocentesis, while helping with early diagnosis. It was emphasized that exchange transfusion is necessary when postnatal bilirubin reaches the exchange threshold.^3^ This was practiced in this case where two double-volume exchange transfusions were given within 24 hours of birth. A 2017 study reported two cases of Rh isoimmunization that were managed non-invasively by using IVIG and phototherapy instead of exchange transfusion.^4^ The IVIG given in this case reduced the need for repeated exchange transfusion by inhibiting antibody-mediated haemolysis. Late onset of severe neonatal anaemia at 45 days postnatally is a unique finding of this case. This highlights the need for ongoing haematological monitoring. IUT is usually a safe procedure with limited complications.^5,6^ Jiandani et al.^1^ mentioned that bone marrow suppression following IUT can lead to late-onset anaemia, though it is not frequently reported. Additionally, a case of postnatal hyperferritinemia and cytomegalovirus infection following multiple IUTs,^7^ requiring close monitoring but no iron chelation therapy, was reported. While our case did not experience such complications, the late-onset anaemia highlights the importance of long-term postnatal haematological surveillance. Along with timely IUT, post-IUT monitoring is of paramount importance.^8^ Similarly, Pares et al.^9^ reported excellent perinatal survival following intrauterine transfusion in a contemporary cohort of Rh-alloimmunized pregnancies, although most fetuses required multiple transfusions before delivery, in contrast to the single late transfusion performed in our patient.

The present case is noteworthy for the successful management of severe fetal anaemia detected late in the third trimester using a single IUT, followed by a favourable long-term neonatal outcome despite severe postnatal haemolytic disease requiring two exchange transfusions and subsequent late-onset anaemia. Unlike many reported cases^2,8,10^ that require serial IUTs because fetal anaemia develops earlier in gestation, our patient presented relatively late at 32 weeks, allowing correction of fetal anaemia with a single well-timed transfusion before delivery. This highlights that individualized management based on gestational age and close fetal surveillance may reduce the need for repeated invasive procedures in carefully selected patients.

The American College of Obstetricians and Gynaecologists (ACOG) Practice Bulletin No. 192, reaffirmed in 2024,^11^ recommends MCA-PSV Doppler as the preferred non-invasive modality for surveillance of pregnancies complicated by red-cell alloimmunization. An MCA-PSV exceeding 1.5 multiples of the median (MoM) is highly predictive of moderate-to-severe fetal anaemia, with a reported sensitivity approaching 100% and a false-positive rate of approximately 12% before the first intrauterine transfusion. The timing of the final intrauterine transfusion and delivery should be individualized according to gestational age, recurrence of fetal anaemia, and overall fetal condition. Management in our patient was consistent with these principles, with Doppler-guided diagnosis, timely intrauterine transfusion at 33 weeks, and planned delivery at 35 weeks and 4 days following multidisciplinary assessment.

## 4. CONCLUSION

Early identification and close surveillance of pregnancies complicated by Rh alloimmunisation are crucial for optimising fetal outcomes. Routine antibody screening and the use of middle cerebral artery (MCA) Doppler as a reliable, non-invasive tool for detecting fetal anaemia allowed timely intervention. In selected cases, appropriately timed intrauterine transfusion can significantly reduce perinatal morbidity and mortality. Delivery planning in a tertiary care setting with access to neonatal intensive care and transfusion services is essential. This case illustrates that timely identification of severe fetal anaemia using MCA Doppler, followed by a carefully timed single intrauterine transfusion in late gestation, can lead to favourable perinatal outcomes even in previously complicated Rh-alloimmunized pregnancies. Despite requiring intensive neonatal management including exchange transfusions, IVIG, and phototherapy, the infant achieved hematologic stabilization and demonstrates normal growth and neurodevelopment at 15 months of age. Structured long-term follow-up remains essential to detect delayed anaemia and optimize outcomes. Multidisciplinary care in a tertiary-level facility is essential for achieving favourable maternal and neonatal outcomes.

## ACKNOWLEDGMENT

The authors thank the Department of OBG, Manipal Hospitals Mangalore for their cooperation.

## AUTHORS’ CONTRIBUTION

All authors contributed equally in data collection and analysis, manuscript drafting and editing.

## CONFLICT OF INTEREST

The authors declare no conflict of interest.

## DISCLOSURE OF AI USE

The authors used AI tools, such as Grammarly and ChatGPT for grammar correction and language improvement.

## DATA AVAILABILITY

The data can be obtained from primary author on due request.

## FUNDING

The author(s) received no financial support for the research, authorship, and/or publication of this article.

## ETHICAL APPROVAL

Institutional approval was not required for the publication of a single case report, as per hospital policy.

## PATIENT CONSENT

Written informed consent for the publication of this case report and accompanying images was obtained from the patient’s parent.
